# Laparoscopic excision of a retroperitoneal mucinous cystic neoplasm: A case report

**DOI:** 10.1016/j.ijscr.2019.07.010

**Published:** 2019-07-19

**Authors:** John Lung, Allison Gracey, Abigail Rosales, Eva Bashover, Alan Sbar, M. Haris Nazim, Ferdinand Rico

**Affiliations:** a Department of Surgery, Texas Tech University Health Sciences Center, 1400 S Coulter, Amarillo, TX, 79106 USA; b Amarillo Pathology Group and Physicians Preferred Laboratory, 1301 S Coulter St #400, Amarillo, TX, 79106 USA

**Keywords:** Mucinous cystic neoplasm, Laparoscopic removal, Case report

## Abstract

**Introduction:**

A 22-year-old female presented with a large abdominal mass that was revealed to be a primary retroperitoneal mucinous cystadenoma.

**Presentation of Case:**

A 22-year-old female presented with a two day history of bloating, mid-epigastric pain, and nausea without vomiting. A CT scan of her abdomen/pelvis showed a large left retroperitoneal mass, possibly a mesenteric cyst. The patient underwent laparoscopic surgery for mass excision. Once the cystic mass was completely dissected laparoscopically, it was placed in a large endobag. The fluid was then aspirated while in the bag to decompress and then completely taken out through a port site. She was discharged the following day. Final pathology revealed a benign mucinous cystadenoma/cystadenofibroma of mesenteric origin.

**Discussion:**

Primary retroperitoneal mucinous cysts are rare occurrences and benign mucinous cystadenomas are the rarest subtype. We use an innovative laparoscopic technique with complete excision of the cyst without spillage of content, thus preventing possible seeding in case of malignancy.

**Conclusion:**

There is some previous caution in using a laparoscopic approach for cystic masses due to potential seeding intra-operatively, in case of fluid spillage of a possible malignant neoplasm. We show through our case that it is possible to efficiently and safely use such an approach.

## Highlights

• Primary retroperitoneal mucinous cystic neoplasms are rare.
• Due to potential seeding intra-operatively, laparoscopic removal was avoided.
• Our case showed efficient and safe use of a laparoscopic approach.
• Surgeons must plan for every cyst to be malignant when planning for removal.
• With a laparoscopic approach, care is required when aspirating the cyst in vivo.

## 1 Introduction

Mucinous cystic neoplasms originate from the ovary and other extra-ovarian sites including the pancreas, mesocolon, mesentery, or any location in the retroperitoneum. [[1], [2], [3], [4]] Most neoplasms are found in the left or right lateral retroperitoneal space [5]. Primary retroperitoneal mucinous cystic neoplasms are broken down into three different categories: benign mucinous cystadenoma, borderline mucinous cystadenoma, and malignant mucinous cystadenoma [6]. Overall, primary retroperitoneal mucinous cystic neoplasms are rare. We report on a case of a retroperitoneal mucinous cystic neoplasm laparoscopically removed with documented histopathology and cytology.

## 2 Presentation of case

**Fig. 1:**
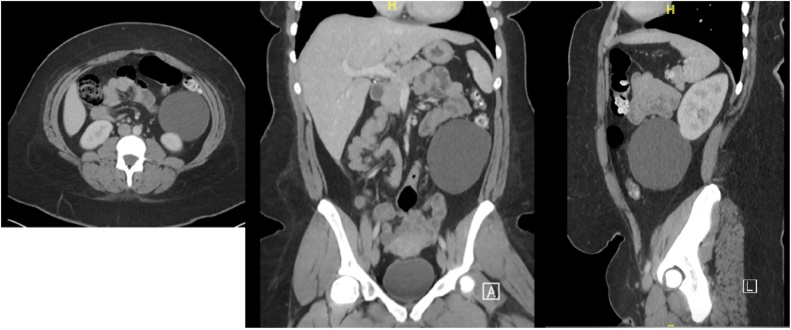
CT scan of mass measuring 8.0 × 8.4 x 9.4 cm: axial, coronal, and sagittal views.

The patient was a 22-year-old female who presented to the emergency department with a two day history of bloating, mid-epigastric pain, and nausea without vomiting. The pain was mild, non-radiating, and unrelated to food intake. She had a past surgical history of ventral hernia repair 6 months prior and laparoscopic cholecystectomy 1 year prior. A CT scan of her abdomen and pelvis showed a large cyst partially abutting the inferior pole of the left kidney that was not clearly renal in origin (Fig. 1). The cyst spatially appeared to be within the retroperitoneum, but possibly represented a mesenteric cyst.

**Fig. 2:**
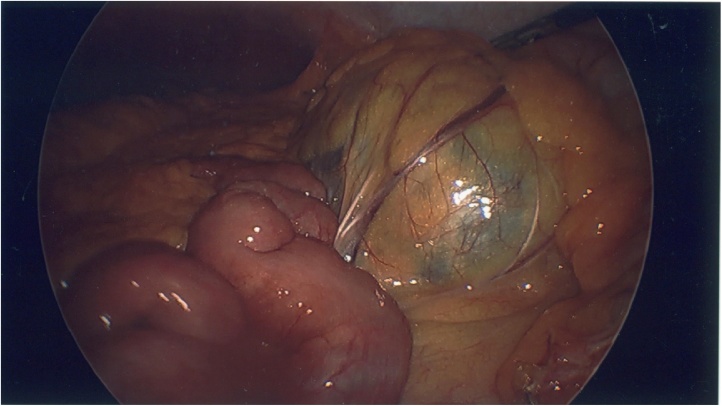
Laparoscopic view of mass prior to excision.

**Fig. 3:**
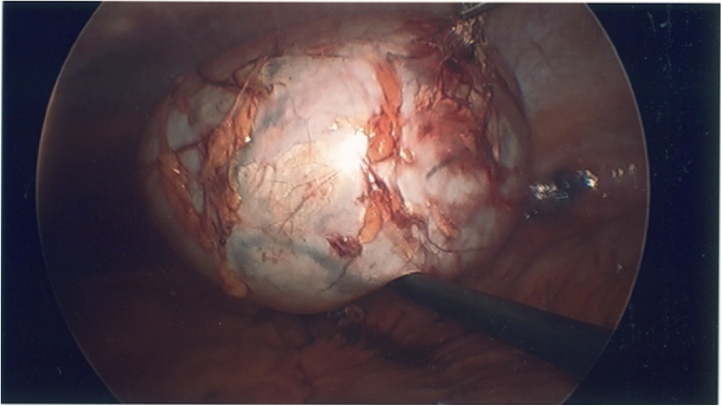
Dissected view of the mesenteric mucinous cystic neoplasm.

**Fig. 4:**
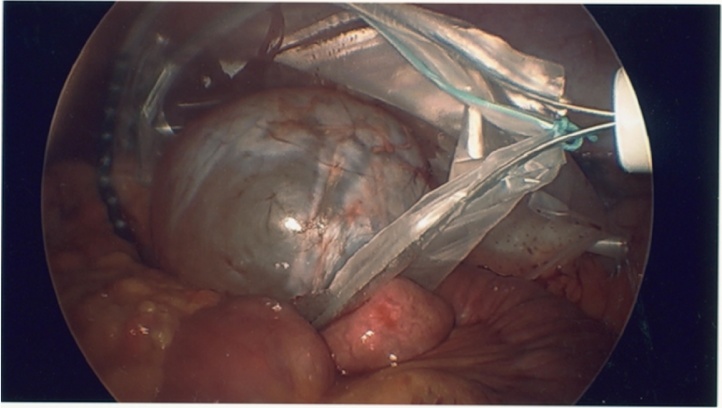
Laparoscopic specimen retrieval bag in use to remove the cystic mass through a 15 mm trocar site.

The mass was diagnosed as non-emergent although the patient elected to have surgical management scheduled for the next day due to her increasing level of pain. The surgical plan was for a laparoscopic excision of mass, possible open, with possible bowel resection. In the operating room, after access to the peritoneal cavity was obtained using an optical bladeless trocar with added ports, lysis of multiple adhesions from previous cholecystectomy and hernia repair was safely done. The greater omentum was transposed superiorly over the stomach and the small bowel was reflected to the right. The left retroperitoneum was widely exposed. The mass was then observed to be retroperitoneal in nature, abutting the left colon. Excision of the mass was done meticulously using Harmonic scalpel and blunt grasper dissection. Bleeding was negligible. The left colon abutting the mass was evaluated thereafter and noted without ischemic changes. The mass was caught with a 15 mm endobag, needle decompression of cyst fluid was done while in the bag, and the bag was delivered out through a 15 mm trocar site (Figs. 2–4). A sample of the cyst wall was then sent for histopathological examination and the aspirated fluid was sent for cytology.

**Fig. 5:**
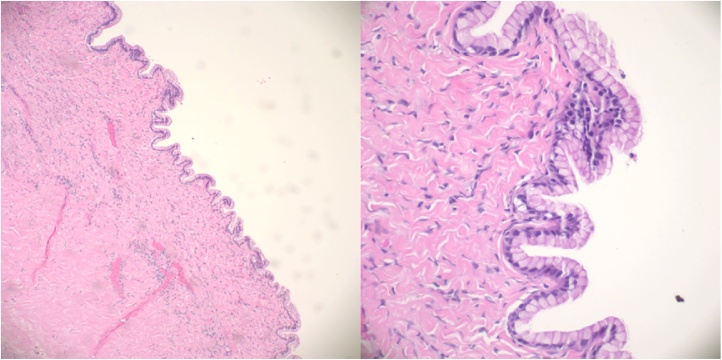
(left) 10x, Low-power microscopic view of cyst highlighting mucinous epithelium without cytologic atypia or architectural complexity. The subepithelial tissue was predominantly fibrous, without diagnostic ovarian-like stroma. (right) 60x, High-power microscopic view of cyst highlighting bland mucinous epithelium.

CEA, CA 125 and tissue pathology was ordered. The CEA and CA-125 values were 1.0 ng/ml and 10.2 units/mL. The final pathology report revealed benign mucinous epithelium without significant cytologic or architectural atypia in a background of fibrous tissue, consistent with a benign mucinous cystic neoplasm of mesenteric origin (Fig. 5).

The patient was transferred to the surgical floor postoperatively, remained uneventful and was discharged the following day.

## 3 Discussion

Mucinous cystadenomas are located in a variety of anatomical regions such as the ovaries, the pancreas, and in the retroperitoneum. Primary retroperitoneal mucinous cysts, as seen in this patient, are rare occurrences. Similar to previous cases, due to the cystadenoma location in the retroperitoneum and the appearance of normal ovaries, it is considered to be a retroperitoneal primary. The origin of these extraovarian mucinous cystadenomas is not clear. One theory claims that the extraovarian cystadenomas are a result of ectopic ovarian tissue or the overgrowth of mucinous epithelium from a teratoma. The theory with major acceptance is that of mucinous metaplasia to an invagination of the peritoneal mesothelial layer resulting in cyst formation. This theory has better recognition versus the ectopic ovarian tissue theory due to the occurrence in males as well. There are three subtypes to these primary retroperitoneal cysts: mucinous cystadenoma, mucinous cystic tumor of borderline malignancy, and mucinous cystadenocarcinoma. [7]

Case reports of mucinous cystadenoma with an origin from the mesentery are very rare with 19 case reports, 2 in males. [4] Of the 19 case reports, 38% are malignant in nature. For this reason, surgeons must plan for every cyst to have malignant potential when planning for cyst removal. Known risk factors for these growths are gender (more common in females) and age (32 ± 13 years). Often biochemistry and hematological studies are within normal ranges in these mucinous cyst cases [2]. Our patient’s CEA of 1.0 ng/mL and CA-125 of 10.2 units/mL are both within normal limits. In our case, our low CEA level is predictive of non-malignancy but did not predict a benign mucinous cystic neoplasm.

One report of two cases uses a laparoscopic surgical approach for simple lymphatic cysts of the mesentery. [8] Other previous case reports defer from the use of laparoscopic surgery and intraoperative aspiration due to large size or due to potential malignant seeding that may lead to later development of pseudomyxoma peritonei [2,9]. This is a prudent precaution with any mass that shows discernable malignant features prior to surgery, such as solid component, or invasion of surrounding structures. However, in this case, there is use of an innovative laparoscopic technique of placing the cystic mass into an endobag still inside the abdominal cavity and aspirating the cyst through a 15 mm trocar site. This technique prevents conversion to an open approach and possible seeding of the cystic contents. Consideration of beginning with a laparoscopic approach with such abnormal cystic growths should be made prior to using open approach due to lower complication rates and faster recovery for the patient [10]. In the event of difficult dissection or firm adherence, conversion to open surgery remains a safe option.

## 4 Conclusion

Primary retroperitoneal mucinous cystic neoplasms rarely occur. We report an innovative laparoscopic removal of a mesenteric mucinous cystic neoplasm with steps to prevent spread if the neoplasm was malignant. A laparoscopic approach that prevents possible seeding should be considered for removal of primary retroperitoneal mucinous cystic neoplasm in the future.

This case report is reported in line with the SCARE criteria [11].

## Declaration of Competing Interest

The authors declare that they have no competing interests. We have no personal or financial conflicts of interest related to the preparation and publication of this manuscript.

## Source of Funding

This research did not receive any specific grant from funding agencies in the public, commercial, or not-for-profit sectors.

## Ethical approval

No ethical approval is required by our institution for the case study presented and submitted.

## Consent

Written informed consent was obtained from the patient for publication of this case report and accompanying images. A copy of the written consent is available for review by the Editor-in-Chief of this journal on request.

## Author contribution

John Lung BS: Data collection, review of literature, co-author of entire manuscript, approval of final manuscript

Allison Gracey BS, BA: Data collection, co-author of case description, approval of final manuscript

Abigail Rosales MBA: Data collection, co-author of discussion, approval of final manuscript

Eva Bashover MD: Conception and design, co-author of case description, critical review of the article, approval of final manuscript

Alan Sbar MD, FACS: Conception and design, critical review of the article, approval of final manuscript

M. Haris Nazim MD, FACS: Conception and design, critical review of the article, approval of final manuscript

Ferdinand Rico MD, FACS: Conception and design, supervisor, co-author of entire manuscript, approval of final manuscript

## Registration of research studies

This is a case report and is not a first-in-man study, therefore registration is not required.

## Guarantor

Ferdinand Rico MD, FACS

## Provenance and peer review

Not commissioned, externally peer-reviewed.
